# Pituitary T-lymphoblastic lymphoma combined with pituitary adenoma: a rare case report

**DOI:** 10.3389/fonc.2026.1704451

**Published:** 2026-04-27

**Authors:** Xiaoyu Chen, Long Cheng, Zhiping Li, Guowei Li

**Affiliations:** 1 Department of Hematology, Huizhou Central People’s Hospital, Huizhou, Guangdong, China; 2 Department of Urology, The Affiliated Huizhou Hospital, Guangzhou Medical University, Huizhou, Guangdong, China; 3 Department of Neurosurgery, Huizhou Central People’s Hospital, Huizhou, Guangdong, China

**Keywords:** case report, central nervous system lymphoma, pituitary, pituitary neuroendocrine tumor (Pit-NET), T lymphoblastic lymphoma

## Abstract

**Background:**

T-cell lymphoblastic lymphoma (T-LBL) is a highly aggressive malignancy that originates from immature precursor T lymphocytes and is characterized by rapid progression. The typical clinical manifestations of T-LBL include superior vena cava syndrome and respiratory compression symptoms such as cough and dyspnea caused by large anterior mediastinal masses. Primary pituitary T-LBL is exceptionally rare, with only seven cases reported worldwide (including the case described in this study).

**Case presentation:**

This article presents the case of an elderly female patient who presented with headaches and progressive visual deterioration. Brain MRI revealed a sellar/suprasellar mass, while whole-body PET/CT imaging revealed a tumor confined to the central nervous system. Laboratory tests for blood parameters and pituitary hormone levels were normal. Following neuroendoscopic tumor resection, pathological examination and immunohistochemical analysis confirmed a diagnosis of pituitary T-LBL coexisting with pituitary adenoma. The patient’s headaches and visual impairment were resolved postoperatively. Subsequent chemotherapy with high-dose methotrexate (HD-MTX), temozolomide, and liposomal doxorubicin effectively controlled the disease.

**Conclusions:**

This case highlights the rarity of concurrent sellar lymphoma and pituitary adenoma and summarizes previously reported cases of primary pituitary T-LBL to provide clinical diagnostic and therapeutic insights into this rare disease.

## Background

T-cell lymphoblastic lymphoma (T-LBL) is a highly aggressive malignant tumor that originates from immature precursor T lymphocytes. Classically, the disease primarily manifests as a large anterior mediastinal solid mass (accounting for approximately 70%-94% of cases), while clinical manifestations typically take the form of compression effects such as superior vena cava syndrome, dyspnea, and chest tightness caused by the mediastinal mass. Among all pituitary tumors, pituitary lymphoma is exceptionally rare, consisting of less than 0.1% of sellar region tumors. As of early 2025, over 60 cases of primary pituitary lymphoma (PPL) have been reported worldwide (1–5), with the vast majority derived from B lymphocytes. Recent reports have further expanded the spectrum of pituitary lymphoma, including rare subtypes such as intravascular large B-cell lymphoma (6), HIV-associated pituitary lymphoma (5). Including the present case, only seven cases of primary pituitary T-LBL have been reported in the literature to date, with the six previously described cases serving as the context for our report. T-cell-origin pituitary lymphomas are exceedingly uncommon, with only 10 documented cases to date, including 7 cases of primary pituitary T-LBL (7–12) and 3 cases of NK/T-cell lineage (4, 13, 14). Notably, some patients develop pituitary lymphoma either simultaneously with pituitary adenoma resection or following adenoma removal (ranging from 4 to 25 years postresection) (12).

According to previous studies, the average age of onset of pituitary lymphoma among adults is 58 years, with a male-to-female ratio of 1.12:1. Most patients with pituitary lymphoma in the sellar/parasellar region present with pituitary insufficiency (72%), headache (56%), diplopia (39%), diabetes insipidus (DI) (39%), visual loss (28%), and fever (22%) (13). All previously reported cases of pituitary T-cell lymphoma also presented with aggressive radiological features accompanied by visual loss or cranial nerve palsy. Given the clinical and radiological similarities between pituitary lymphoma and pituitary adenoma, the diagnosis cannot be based solely on clinical manifestations or imaging findings; cytological examinations and immunohistochemistry (IHC) are essential for confirming the lymphoid origin and evaluating the underlying pituitary cellular background.

This case reports only the seventh reported instance of primary pituitary T-lymphoblastic lymphoma. The mass was initially misdiagnosed as a pituitary adenoma on imaging, but postoperative immunohistochemical staining and a detailed cytological examination confirmed a diagnosis of primary pituitary lymphoma. Pituitary T-LBL is exceedingly rare and has a highly aggressive clinical course, necessitating highly intensive therapeutic approaches. Thus, clarifying the clinical manifestations, radiological features, and immunohistochemical findings of pituitary T-LBL is critical for the early diagnosis and effective treatment of this rare disease.

## Case presentation

### Clinical data

The patient was a 61-year-old female with a history of hypertension. She presented with intermittent headaches for six months and progressive visual decline over the prior two months. She denied a history of fever, weight loss, night sweats, limb weakness, or diplopia. A prior cranial magnetic resonance imaging (MRI) scan at an outside hospital revealed a “sellar and suprasellar mass,” prompting admission to Huizhou Central People’s Hospital for further evaluation and management. The patient had no known history of primary or acquired immunodeficiency, autoimmune disease, or prior immunosuppressive therapy. HIV serology was negative.

Physical examination on admission revealed no palpable superficial lymph nodes, decreased left visual acuity on crude testing, unrestricted bilateral ocular movements, and no hepatosplenomegaly. Laboratory tests revealed normal blood counts, electrolytes, liver and kidney function, and urine osmolality but elevated lactate dehydrogenase (LDH; 260 U/L, reference range: 120–246 U/L). Hormone levels (sex hormones, thyroid hormones, cortisol) were within normal ranges. The levels of tumor markers (carcinoembryonic antigen [CEA], alpha-fetoprotein [AFP]) and immunological parameters (complements C3 and C4, immunoglobulin G [IgG], antinuclear antibody [ANA] profile) were unremarkable. The patient had no history of radiotherapy or chemotherapy.

**Figure 1:**
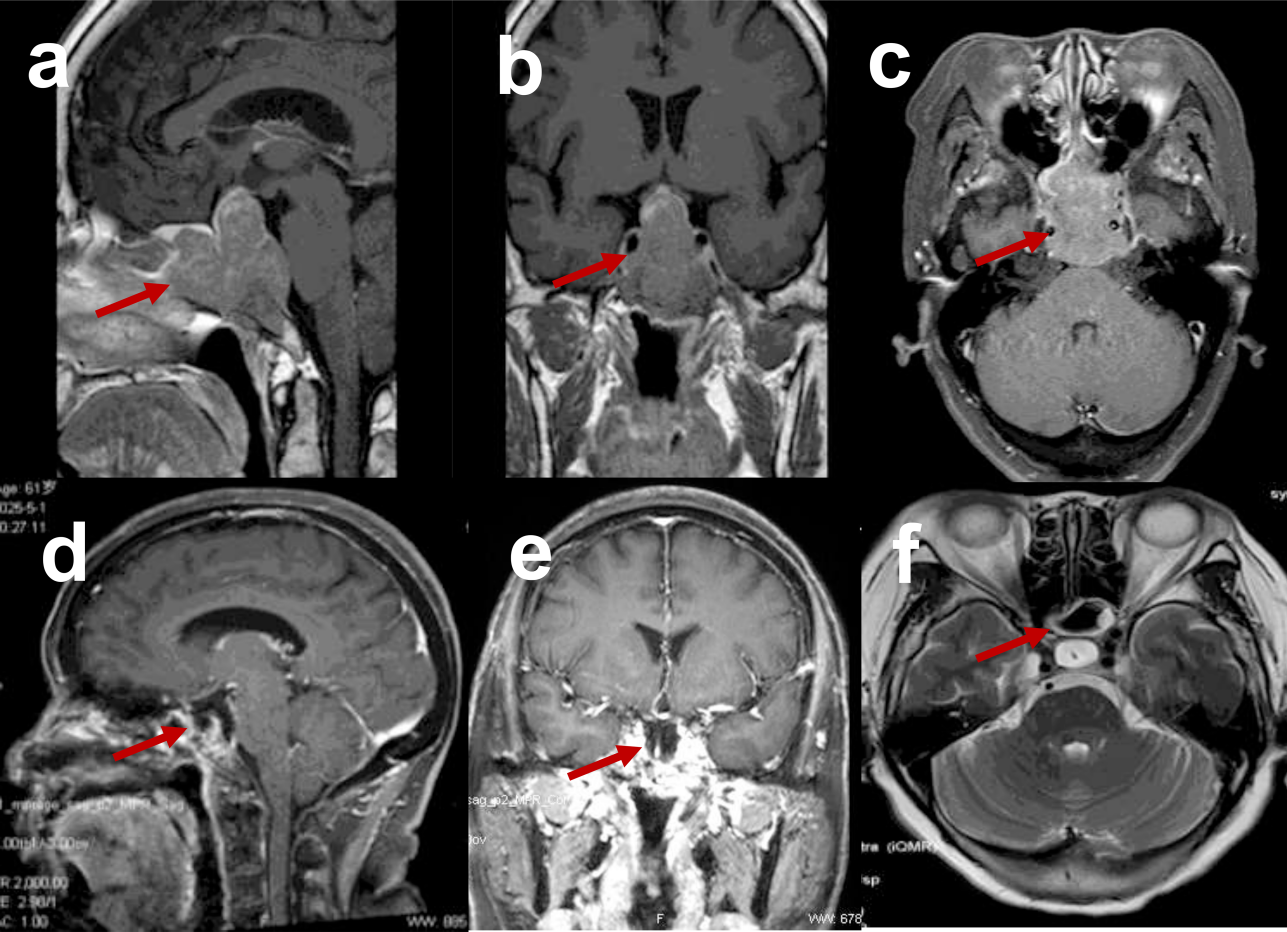
Comparison of preoperative and postoperative contrast-enhanced MRI scans of the brain. Preoperative MRI demonstrated a well-defined, irregular mass within the sellar/suprasellar regions and sphenoid sinus. T1-weighted images showed a homogeneous isointense mass with internal nodular hyperintense foci. The lesion was primarily located within the sellar region, obscuring the pituitary gland and pituitary stalk. It exhibited a drill-like growth pattern, accompanied by osteolytic destruction of the sphenoid sinus wall and skull base. The optic chiasm was displaced superiorly due to compression from the mass **(a–c)**. Seven months after surgery and chemotherapy, brain MRI revealed no tumor **(d–f)**.

MRI revealed an irregular, slightly hyperintense mass in the sellar/suprasellar region and sphenoid sinus measuring approximately 40 mm × 34 mm × 46 mm (anteroposterior × transverse × craniocaudal). The mass exhibited well-defined but irregular margins, a homogeneous density, and nodular, hyperintense foci. The lesion was primarily located in the sellar region, obscuring the pituitary gland and stalk, and demonstrated a drill-like growth pattern, destruction of the sphenoid sinus wall and skull base, and upward displacement of the optic chiasm. No abnormal densities were observed in the brain parenchyma, ventricular system, or cisterns, and the midline structures remained central (**Figures 1a–c**).

**Figure 2:**
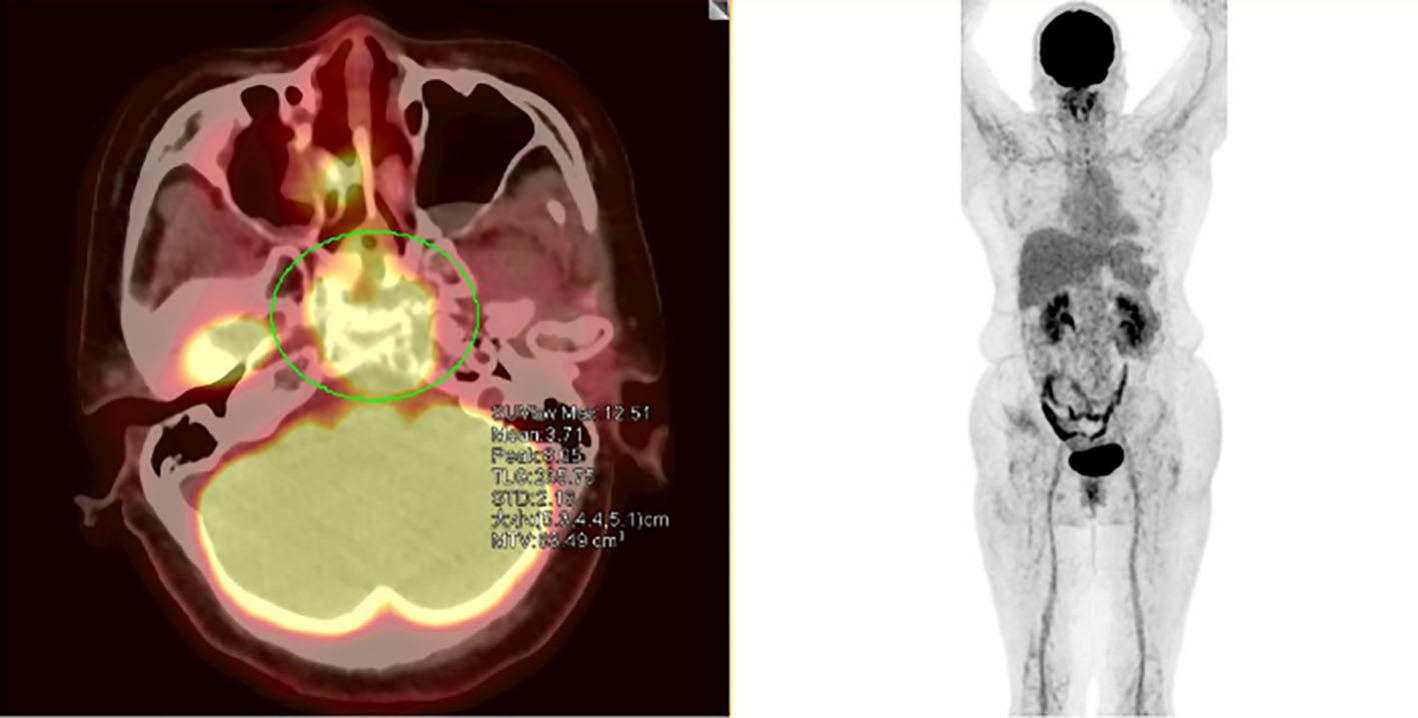
Postoperative PET/CT. PET/CT findings: Increased FDG uptake (SUVmax=12.51) in the sellar region with sphenoid/clival destruction.

Positron emission tomography/computed tomography (PET/CT) imaging revealed increased fluorodeoxyglucose (FDG) uptake in the sellar/suprasellar region, sphenoid sinus, and right middle nasal meatus (SUVmax = 12.51), with a maximum cross-sectional dimension of 32 mm × 43 mm. An irregular soft tissue density and osteolytic destruction of the sphenoid sinus wall and clivus were noted, and no abnormal hypermetabolic activity was observed elsewhere (**Figure 2**). Bone marrow flow cytometry revealed no abnormalities, and bone marrow biopsy pathology with IHC confirmed no evidence of T-lymphoma cell infiltration.

### Surgery and pathology

**Figure 3:**
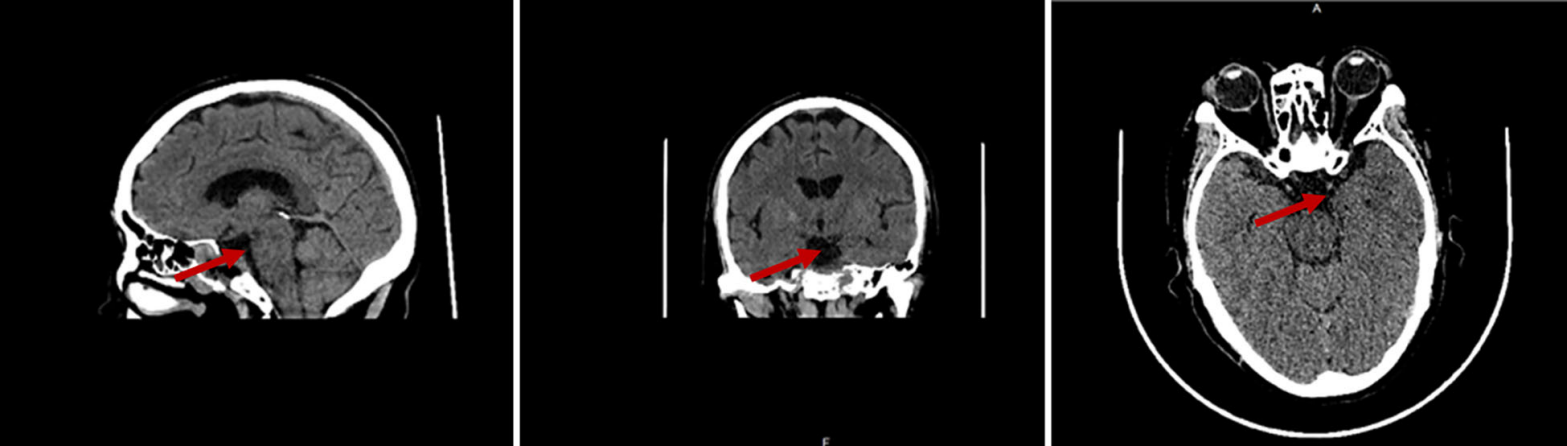
First postoperative follow-up. On postoperative day 5, plain cranial CT scan with three-dimensional reconstruction shows: Postoperative changes in the suprasellar-sellar region; the previously observed mass shadow is no longer visible, with localized irregularity of the adjacent bone.

The patient underwent neuroendoscopic transnasal-sphenoidal resection of the pituitary lesion (via a neuroendoscopic right nostril-sphenoidal approach for sellar pituitary tumor resection). Surgical procedure: Intraoperatively, the sellar floor appeared largely intact. The bone of the sellar floor was thinned with an eggshell technique, exposing the sellar dura. Upon incision of the latter, slightly reddish pituitary tissue was observed posteroinferior to the tumor. The tumor itself appeared yellowish and was firm in texture. Using a curette, the lesion was excised along the inner tumor capsule, starting from the right aspect and then proceeding to the left, posterior, and anterior aspects. Upon completing the anterior resection, the diaphragma sellae were visualized, and residual tissue was carefully curetted under the diaphragma. Exploration confirmed that there was no substantially residual abnormal tissue. The procedure concluded uneventfully. On postoperative day 5, cranial CT showed no evidence of the original suprasellar and sellar mass lesions (**Figure 3**).

**Figure 4:**
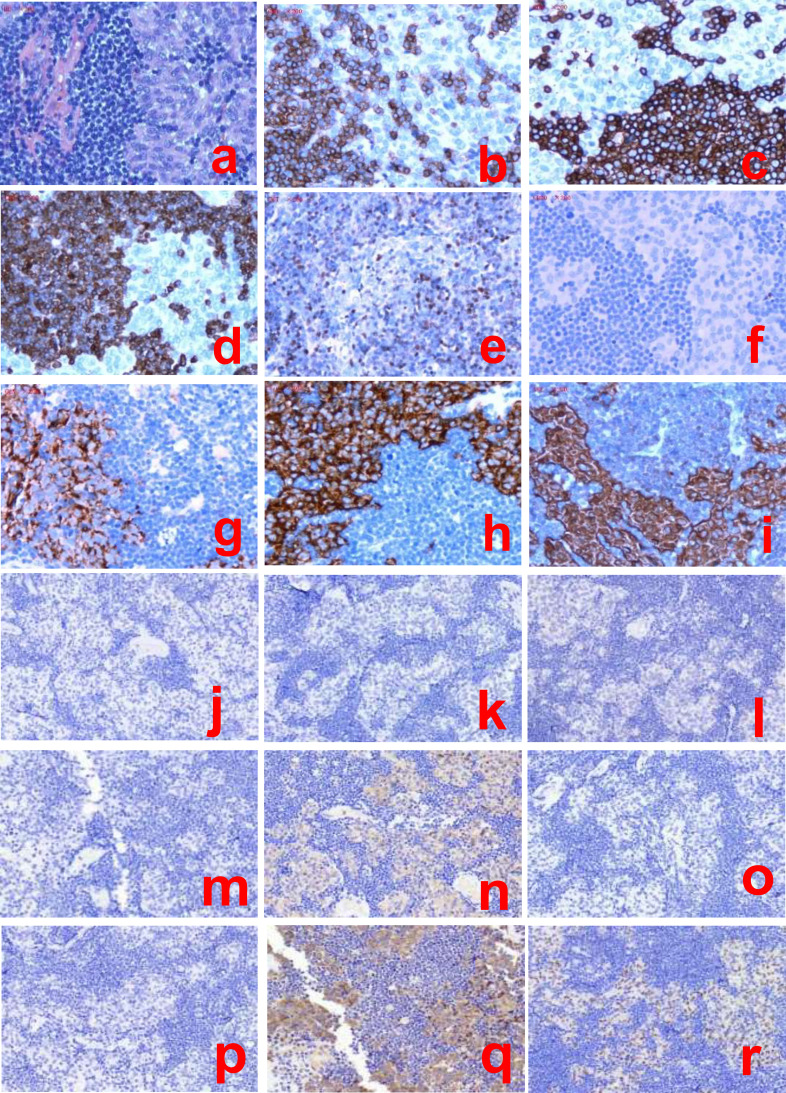
Immunohistochemistry of the lymphoma and pituitary adenoma (**(a)**: H&E staining; **(b–i)**: CD3, CD4, CD8, TdT, CD20, CgA, Syn, NSE).(**(j–r)**: ACTH, FSH, hGH, LH, PRL,TSH, PIT-1, SF-1, T-PIT). Tumor cells are positive for T-cell markers (CD3, CD4, CD8, and TdT),and negative for B-cell markers (CD20). Pituitary adenoma cells express neuroendocrine markers (CgA, Syn, and NSE). hGH (−), PRL (scant weak+), ACTH (−), TSH (−), LH (−), FSH (−), PIT1 (−),SF-1 (−),T-PIT (focal weak+).

Postoperative pathological examination of the excised tissue revealed a sellar and suprasellar mass consistent with T-LBL concurrent with pituitary adenoma. Immunohistochemistry (IHC): Lymphoma component: CK(−), EMA(−), Vim(−), GFAP(−), CD3(+), CD5(+), CD20(−), PAX-5(−), CD30(−), CD99(+), TdT (focal weak +), CD34(−), CD4(+), CD8 (focally positive), GrB(−), CD56 (–), and Ki-67 (90%); pituitary adenoma component: NSE(+), CgA(+), Syn(+); pituitary hormone profile: GH(−), PRL(focal weak+), ACTH(−), TSH(−), LH(−), FSH(−), consistent with a non-functioning null cell pituitary neuroendocrine tumor (Pit-NET) according to the WHO 5th edition (2022) classification. Transcription factor analysis showed Pit-1(−), T-pit(focal weak+), SF-1(−). *In situ* hybridization: EBER (−) (**Figure 4**).

### Treatment and follow-up

The patient developed pituitary insufficiency postoperatively and was started on glucocorticoid replacement therapy. Hydrocortisone (30 mg once daily) was administered for glucocorticoid replacement therapy, and pituitary hormone levels were monitored regularly with the medication dosage adjusted according to the test results. Concurrently, the patient underwent chemotherapy, consisting of HD-MTX (3.5 g/m² on day 1), liposomal doxorubicin (40 mg/m² on day 2), and temozolomide (200 mg daily on days 1–5), which were administered every 28 days for a total of 6 cycles. Lumbar puncture with intrathecal injection was performed, revealing normal cerebrospinal fluid (CSF) pressure and unremarkable routine, biochemical, and flow cytometry CSF findings. Postoperative cranial MRI revealed no residual mass, and the patient reported resolution of her headaches and restored vision. Follow-up MRI after two chemotherapy cycles confirmed that there was no recurrent lesion in the pituitary region, while contrast imaging revealed short T1 and long T2 signal intensities at the surgical margins and no abnormal enhancement (**Figures 1d–f**). Follow-up commenced in October 2024 (immediately postoperatively), and as of March 2026, the patient has been followed for 17 months and remains in complete remission (**Table 1**).

**Table 1:** Timeline of diagnosis, treatment and follow-up for pituitary T-LBL with pituitary adenoma.

Time point	Clinical event	Examinations/interventions	Key outcomes/findings
6 months pre-admission	Initial symptom onset	None (no medical consultation)	Intermittent headache; no impact on daily activities
2 months pre-admission	Symptom progression	Cranial MRI (peripheral hospital)	Progressive left visual decline; MRI indicated sellar/suprasellar mass
Admission day	Baseline assessment	Physical exam, lab tests, pituitary hormones, tumor markers, HIV serology, bone marrow tests	Left visual acuity decreased; LDH 260 U/L (mild elevation); all others normal; HIV⁻
3 days post-admission	Imaging assessment	Contrast-enhanced cranial MRI, whole-body PET/CT	40×34×46 mm sellar/suprasellar/sphenoid sinus mass; SUVmax=12.51; CNS-confined tumor
7 days post-admission	Surgical intervention	Neuroendoscopic transnasal-sphenoidal resection (right nostril approach)	Successful complete tumor resection; headache relieved post-op day 1; no complications
5 days post-op	Early post-op evaluation	Cranial CT	No residual mass; no intracranial hemorrhage/edema
7 days post-op	Diagnosis + replacement therapy	Pathological/IHC/ISH tests; hydrocortisone (30 mg/day) initiated	Diagnosed as pituitary T-LBL + non-functional Pit-NET (Ki-67 90%); hypopituitarism managed
2 weeks post-op	First-line chemotherapy init	Cycle 1: HD-MTX + liposomal doxorubicin + temozolomide (28-day intervals); LP + intrathecal injection	Normal CSF; mild fatigue only; slight visual improvement
2 months post-op (2 chemo cycles)	Mid-chemo evaluation	Contrast-enhanced MRI, pituitary hormone/hepatorenal function recheck	No tumor recurrence; left vision significantly recovered; headache resolved; good tolerance
6 months post-op (6 chemo cycles completed)	Post-chemo evaluation	Contrast-enhanced MRI, PET/CT, LDH/pituitary hormone recheck	No recurrence/dissemination; LDH normal; vision nearly recovered; hydrocortisone continued
17 months post-op (Mar 2026)	Last follow-up	Contrast-enhanced MRI, pituitary hormone/hepatorenal function recheck	Complete remission; good treatment adherence; normal daily life resumed

## Discussion

Pituitary adenomas are the second most common type of primary brain tumor, with an estimated asymptomatic prevalence of approximately 10% in the general population (15). The most common pituitary adenomas, in descending order, are prolactinomas, nonfunctioning adenomas, growth hormone-secreting adenomas, adrenocorticotropic hormone-secreting adenomas, and thyroid-stimulating hormone-secreting adenomas (15). In contrast, pituitary lymphomas are exceedingly rare among all sellar tumors, accounting for less than 0.1% of all cases. Retrospective case analyses have reported a B-cell to T-cell origin ratio of 5.5:1 for pituitary lymphomas, mirroring the distribution observed in peripheral lymphomas (2). Notably, pituitary lymphoma is only one of many rare sellar lesions that may mimic pituitary adenoma on imaging (16). Almomen et al. reported a case of sellar-suprasellar pituitary lymphoma that was initially misdiagnosed as a prolactinoma due to elevated prolactin levels and typical imaging features (3). Including intravascular lymphoma and chronic lymphocytic leukemia, should also be considered as rare causes of sellar masses presenting with hypopituitarism (17).

Pituitary T-LBL, an even rarer subtype of central nervous system lymphoma, is clinically similar to pituitary adenoma but is more aggressive and has a poorer prognosis. To our knowledge, only 7 cases of primary pituitary T-LBL have been reported in the literature through 2025. This article synthesizes existing cases with the present case to provide a comprehensive summary of this rare entity.

On the basis of existing data from the 7 reported cases of primary pituitary T-LBL, this entity has a marked female predominance (all 7 cases were female), which contrasts with the male predominance reported in the literature on general pituitary lymphomas. The age of onset ranges from 45 to 64 years (approximately middle to elderly ages), with a mean of 58.3 years (**Table 2**). This age distribution suggests a potential association with hormonal changes, such as postmenopausal elevation of gonadotropins and decreased estrogen level.

**Table 2:** Summary of reported primary pituitary T-LBL cases.

Reference	Sex	Age (yrs)	Symptoms	Endocrine findings	Radiology	Treatment	Follow-up
Kuhn et al. (7)	F	67	Oculomotor nerve palsy and amaurosis of the right side	Hypopituitarism	Suprasellar mass with homogeneous density and invasion of clivus	Invasion of right cavernous sinus 6 months after surgery, followed by radiation to sellar region.	Residual tumor after 5 months
Romeike et al. (8)	F	64	Left cranial nerve III paresis, double vision, left eye ptosis, bitemporal hemianopsia	Pituitary insufficiency/hypopituitarism	Contrast-enhancing intra- and suprasellar mass partially encasing left internal carotid artery, causing elevation of optic chiasm and involving left cavernous sinus.	Post-operative methotrexate and external beam radiation.	19 months of stability
Wiens et al. (10)	F	58	Headache, right eye ptosis, cranial nerve III palsy	Pituitary insufficiency/ hypopituitarism, DI	Heterogeneously contrast-enhancing intra- and suprasellar mass displacing optic chiasm and extending into right cavernous sinus.	Leptomeningeal dissemination. Post-operative intrathecal chemotherapy and craniospinal radiation.	Death (meningeal spread)
Gupta et al. (9)	F	55	Headache, bilateral diminution of vision, altered sensorium	Hypopituitarism (LH, TSH), and hyperpituitarism (prolactin, evening cortisol)	Sellar/ suprasellar mass extending into suprasellar cistern, lateral ventricle, encasing Lt ICA, invading optic chiasm and infundibulam	Surgery	Death 2 months after surgery
Sreedhara et al. (11)	F	45	Headache, ptosis, diplopia, blurry vision CN III, IV, VI palsy, V1 numbness	Secondary adrenal insufficiency, hyperprolactinemia, secondary hypothyroidism	llar mass extending into sphenoid and cavernous sinuses and clivus, encasement of internal carotid artery (ICA), abutment of optic chiasm	transsphenoidal resection. HD-MTX, regularly lumbar punctures with intrathecal chemo-therapy. alternating cyclophosphamide, vincristine sulfate, doxorubicin hydrochloride, and dexamethasone and methotrexate-cytarabine.	Partial improvement
Koga et al. (12)	F	51	blurred vision and a visual field defect	slightly elevated prolactin (PRL) and low insulin-like growth factor-1 levels	a sellar mass extending to the suprasellar region, compressing the optic chiasma upward	Two weeks postoperatively, leptomeningeal and pancreatic dissemination occurred. Treatment included cyclophosphamide, doxorubicin, vincristine, dexamethasone, methotrexate, and radiation. Paraplegia developed due to spinal dissemination.	Death (10 months postop)
Current case (2025)	F	61	Headache, visual decline	/	mass in the sellar, suprasellar region and sphenoid sinus	Surgery and HD-MTX, liposomal doxorubicin, and temozolomide	17 months of remission

The neurological manifestations of pituitary lymphoma resemble those of pituitary adenomas, as they depend on the location and extent of spread of the tumor. The most common clinical presentations include hypopituitarism, headache, and visual disturbances such as hemianopia or diplopia. Additional systemic symptoms may include fatigue, weight loss, fever, nausea, and vomiting (2). Acute total ophthalmoplegia may serve as a presenting manifestation of primary pituitary lymphoma (1). If the lymphoma extends to the cavernous sinus or orbital apex, it may cause cranial nerve palsies (most frequently affecting cranial nerves II and III) and retro-orbital pain (18). Notably, endocrine abnormalities such as hypopituitarism and DI are more prevalent in patients with pituitary lymphoma than in those with pituitary adenoma. Hypopituitarism in patients with pituitary lymphoma often manifests with diverse symptoms, including fatigue, muscle weakness, reduced libido, amenorrhea, thirst, and polyuria (13).

Among the 7 reported cases of pituitary T-LBL, the neurological symptoms primarily consisted of visual decline (85.7%), cranial nerve (CN) palsies (100%, most commonly involving CN III, IV, and VI), and headache (71.4%). Endocrine abnormalities predominantly included hypopituitarism (71.4%), and hyperprolactinemia (28.5%, 2 cases). Additional symptoms included diplopia, ptosis, and altered consciousness, the latter of which were likely associated with tumoral invasion or meningeal dissemination.

The patient reported in this case report also exhibited visual decline and headache. Preoperative evaluations revealed normal pituitary function and no evidence of adrenal insufficiency or thyroid dysfunction. Postoperatively, her visual deficits and headaches improved significantly. However, secondary adrenal insufficiency developed after surgery, prompting the initiation of hydrocortisone replacement therapy.

The radiological features of pituitary lymphoma include isointense lesions on T1-weighted imaging (T1WI), hypointense lesions on T2WI, and heterogeneous enhancement. All pituitary masses in these cases exhibited aggressive radiological characteristics, such as invasion of the clivus, sphenoid sinus, or cavernous sinus; erosion of the sellar floor; and encasement of the internal carotid artery. Given that the tumor frequently extended into the suprasellar region, cavernous sinus, and sphenoid sinus in most reported cases (7–9, 19–23), some scholars have suggested that sphenoid sinus mucosal biopsies should be performed for patients whose pituitary masses display aggressive imaging features. Such an approach may help confirm the diagnosis while avoiding the surgical risks associated with pituitary resection (2).In our retrospective analysis of all reported cases of pituitary T-LBL, sellar masses were universally present, frequently invading the cavernous sinus (85.7%), sphenoid sinus (57%), and clivus (57%), and 57.1% of the masses encased the internal carotid artery. Two patients demonstrated meningeal or spinal dissemination, and one patient developed systemic metastasis (pancreatic involvement). On PET/CT imaging, some patients presented with hypermetabolic foci, although differentiation from adenoma remains challenging. In our case, the tumor extended to the suprasellar region, compressed the optic nerves (a previously reported manifestation), and invaded the sphenoid sinus, cavernous sinus, and even the clivus. While endocrine, neurological, and radiological assessments are crucial for diagnosing and managing primary pituitary lymphoma, alone, they are insufficient for distinguishing the disease from pituitary adenoma.

The pathogenesis of primary pituitary lymphoma is poorly understood, but several hypotheses have been proposed:

1. Hormone-Driven Hypothesis: Hormones secreted by pituitary adenomas (e.g., follicle-stimulating hormone [FSH] and adrenocorticotropic hormone [ACTH]) may promote lymphocyte proliferation via hormone receptor-mediated mechanisms. For example, in two reported cases of T-LBL coexisting with FSH-positive adenomas, FSH was hypothesized to activate proliferative signaling pathways through T-cell surface receptors (7, 24).
2. Adhesion Molecule-Mediated Hypothesis: Lymphoma cells may express neural cell adhesion molecules (NCAMs) or integrins, allowing their specific colonization within the pituitary microenvironment (25).
3. Inflammatory Transformation Hypothesis: Chronic lymphocytic hypophysitis may undergo monoclonal transformation, particularly in immunosuppressed or genetically predisposed individuals (26).
4. Surgical or Radiotherapy-Induced Hypothesis: Some patients (e.g., one described in a 2024 case report) developed T-LBL years after pituitary adenoma resection, suggesting that surgical disruption of the blood–brain barrier or localized inflammation may facilitate lymphomagenesis (12).

The gold standard for diagnosing this disease is pathology. Histologically, T-LBL is characterized by diffuse infiltration of small- to medium-sized lymphoblasts with abundant mitotic figures. Immunohistochemically, the tumor cells typically express CD3, CD4, CD8, and TdT and the Ki-67 index is elevated, while tests for B-cell markers (e.g., CD20) are negative. Molecular testing for T-cell receptor (TCR) gene rearrangement can confirm clonality (27). In our patient, the Ki-67 proliferation index was as high as 90%, indicating an extremely high proliferative activity of the tumor cells and being consistent with the highly aggressive features of T-LBL. Regrettably, clonality molecular testing was not performed due to the patient’s financial constraints. However, in clinical and pathological practice, a definitive diagnosis of T-LBL can still be made based on morphological features and key immunophenotypic findings alone, even without performing TCR gene rearrangement testing. Differential diagnoses include other pituitary neoplasms, such as pituitary adenoma, distinguished by positive immunohistochemical staining for pituitary hormones and, typically, reactive lymphocyte infiltration; lymphocytic hypophysitis, characterized pathologically by polymorphic inflammatory cell infiltration and a lack of clonality in lymphocytes; and secondary pituitary involvement by systemic lymphoma, which requires a thorough systemic evaluation to exclude secondary involvement.

Notably, a retrospective analysis revealed that among the 7 cases of pituitary T-LBL, 6 (85.7%) included confirmation of synchronous or sequentially occurring pituitary adenoma (**Table 3**). Three of these cases were identified simultaneously during surgery (synchronous diagnosis), whereas the remaining 3 cases developed pituitary lymphoma 9–25 years after pituitary adenoma resection and had often been misdiagnosed as recurrence of the adenoma. Among these 6 patients, 2 had gonadotropin-positive adenomas, 2 had nonfunctioning adenomas, and 2 had a combined ACTH-secreting adenoma. The concurrent or sequential occurrence of pituitary lymphoma and adenoma suggests that pituitary hormones may promote lymphocyte proliferation and contribute to lymphomagenesis.

**Table 3:** Relationship between pituitary T-lymphoblastic lymphoma and pituitary adenoma.

Case source	Lymphoma-adenoma relationship	Adenoma characteristics (hormone secretion)
Kuhn et al. (7)	Lymphoma recurrence within a pituitary adenoma (25 years postresection)	FSH-positive adenoma
Romeike et al. (8)	Lymphoma arising in a recurrent pituitary adenoma (17 years postresection)	FSH-positive adenoma (5% cells expressed FSH)
Wiens et al. (10)	Coexisting lymphoma and adenoma (synchronous diagnosis)	pituitary ACTH-cell hyperplasia (other hormones variably expressed
Gupta et al. (9)	Coexisting lymphoma and adenoma (synchronous diagnosis)	ACTH/TSH-positive adenoma
Koga et al. (12)	Lymphoma developed 9 years postresection of pituitary adenoma	Nonfunctioning adenoma
Current Case (2025)	Coexisting lymphoma and adenoma (synchronous diagnosis)	Nonfunctioning adenoma

The WHO 5th edition (2022) classification of pituitary tumors now designates pituitary adenomas as pituitary neuroendocrine tumors (Pit-NETs), classified based on transcription factor lineage (Pit-1, T-pit, SF-1) rather than hormone expression alone. This framework better characterizes pituitary tumors and has been applied to the adenoma component in the reported cases. In our case and other reported cases, the non-functioning adenomas were classified as null cell Pit-NETs, negative or focally weakly positive for all anterior pituitary hormones and transcription factors.

Among the 7 reported cases of pituitary T-LBL, partial transsphenoidal resection was performed in most cases to alleviate the mass effect, whereas gross total resection was rarely achievable because the tumor had invaded the cavernous sinus or vascular encasement. Several patients received HD-MTX-based central nervous system penetrating chemotherapy, often combined with cyclophosphamide, vincristine, and other agents (e.g., hyper-CVAD). Additionally, 71.4% of patients underwent whole-brain or sellar radiotherapy (45–50 Gy) to control local residual disease or leptomeningeal dissemination. To date, no evidence-based data supports the use of targeted therapies or hematopoietic stem cell transplantation (HSCT), although these modalities represent potential investigational approaches. Filo et al. recently re-evaluated the role of surgical resection in the management of primary pituitary lymphoma and proposed that surgery should be limited to tissue diagnosis rather than radical resection, given the superior efficacy of chemotherapy-based approaches for this entity (28).

Primary pituitary T-LBL is associated with dismal survival outcomes, as evidenced by the seven reported cases. Of these, three patients died of disease progression or dissemination: two had definitive postoperative survival times of 2 and 10 months (median survival: 6 months), and one succumbed to leptomeningeal spread with no specific survival data available. The other four patients remained alive at last follow-up with variable disease status: one had residual tumor and cavernous sinus invasion at 6 months post-surgery, one achieved 19 months of sustained disease stability after chemo-radiotherapy, one had partial clinical improvement with unreported follow-up duration, and our present case maintained complete remission for 17 consecutive months (Oct 2024–Mar 2026) following neuroendoscopic resection and six cycles of HD-MTX-based chemotherapy. Due to the high proportion of censored data and missing survival information, the median overall survival for this rare disease has not been reached to date. Early diagnosis combined with intensive chemotherapy may alleviate the patient’s symptoms; however, the high probability of recurrence persists, underscoring the need for novel therapeutic strategies.

In summary, we present a case of primary pituitary T-LBL. Although pituitary lymphoma is rare, it should be considered in the differential diagnosis of sellar masses, particularly when neuroimaging suggests invasion of adjacent structures. While most sellar lesions originate from pituitary cells—making hormonal evaluation critical for a diagnosis of adenoma—a definitive diagnosis of pituitary lymphoma relies on immunohistochemical staining and microscopic identification of lymphoblastic tumor cells. In addition to endocrine, neurological, and radiological assessments, prompt immunohistochemical profiling and comprehensive molecular analysis (including clonality testing) are essential for early diagnosis and improving outcomes.

Notably, all reported pituitary T-LBLs occurred in middle-aged to elderly females, and the majority were diagnosed concurrently or sequentially with pituitary adenomas. A subset of these adenomas was gonadotropin-secreting, suggesting that hormonally driven lymphocyte proliferation may contribute to lymphomagenesis—a hypothesis warranting further investigation. Of note, a recent SEER database analysis demonstrated that patients with pituitary adenoma have a significantly increased risk of developing subsequent primary malignancies, particularly lymphatic and hematopoietic cancers (29), which further supports the possible association between pituitary adenoma and lymphoma development. Pituitary T-LBL is a highly aggressive malignancy; while multimodal therapy (surgery, chemotherapy, and radiotherapy) may provide short-term disease control, novel targeted therapies must be explored to address its poor prognosis and high recurrence rates.

## Study limitations

This study has additional inherent limitations that warrant acknowledgment. First, molecular biological profiling of the pituitary lymphoma—including the detection of specific gene mutations and analysis of dysregulated signaling pathways linked to lymphomagenesis—was not conducted. This gap precludes an in-depth investigation into the pathogenic mechanisms of pituitary T-LBL, especially the potential molecular crosstalk between pituitary neuroendocrine tumor (Pit-NET) and T-lymphoblastic lymphoma cells within the sellar microenvironment. Second, as a single-center case report without a multicenter case-control design, the extreme rarity of pituitary T-LBL prevented us from enrolling an adequate sample size for comparative analysis of clinical, radiological and pathological variables. Consequently, independent prognostic factors for this highly aggressive malignancy could not be identified or validated. Third, no long-term follow-up data beyond 17 months are available for the present patient, and the lack of extended follow-up makes it impossible to assess the long-term efficacy of the combined chemotherapeutic regimen adopted and the late recurrence risk of pituitary T-LBL. Fourth, intratumoral immune microenvironment analysis (e.g., immune cell infiltration, cytokine expression) was not performed, which limits the exploration of the immune regulatory mechanisms underlying the coexistence of Pit-NET and T-LBL in the sellar region. Fifth, the present study did not evaluate the potential impact of postoperative hormonal replacement therapy on lymphoma cell proliferation and disease progression, a critical consideration given the hypothesized hormone-driven lymphomagenesis in pituitary T-LBL. Finally, no comparative analysis with other chemotherapeutic or chemoradiotherapeutic regimens was conducted, as there is currently no standardized treatment protocol for pituitary T-LBL, making it impossible to verify the optimality of the therapeutic strategy applied in this case.

## Data Availability

The raw data supporting the conclusions of this article will be made available by the authors, without undue reservation.
